# ProsCan for Men: Randomised controlled trial of a decision support intervention for men with localised prostate cancer

**DOI:** 10.1186/1471-2407-8-207

**Published:** 2008-07-24

**Authors:** Suzanne K Chambers, Megan Ferguson, RA Gardiner, David Nicol, Louisa Gordon, Stefano Occhipinti, Joanne Aitken

**Affiliations:** 1 Viertel Centre for Research in Cancer Control, Cancer Council Queensland, Brisbane, Australia; 2 School of Psychology, Griffith University, Brisbane, Australia; 3 National Executive, Australian Prostate Cancer Collaboration, Melbourne, Australia; 4 Department of Surgery, University of Queensland, Brisbane, Australia; 5 Department of Urology, Princess Alexandra Hospital, Brisbane, Australia; 6 Cancer and Population Health Studies, Queensland Institute of Medical Research, Brisbane, Australia; 7 School of Population Health, University of Queensland, Brisbane, Australia

## Abstract

**Background:**

Prostate cancer is the most common male cancer in the Western world but is highly heterogeneous in disease progression and outcomes. Consequently, the most substantial morbidity may actually arise from the adverse psychosocial impact of distress in decision-making and long term quality of life effects such as impotence. This paper presents the design of a randomised controlled trial of a decision support/psychosocial intervention for men newly diagnosed with localised prostate cancer.

**Methods/Design:**

350 men per condition (700 men in total) have been recruited after diagnosis and before treatment through urology private practices and hospital outpatient clinics and randomised to 1) a tele-based nurse delivered five session decision support/psychosocial intervention or 2) a usual care control group. Two intervention sessions are delivered before treatment that address decision support, stress management and preparation for treatment. Three further sessions are provided three weeks, seven weeks and five months after treatment that focus on adjustment to cancer, problem solving and coping with treatment side effects. Participants are assessed at baseline (before treatment) and 2, 6, 12, 24 and 36 months post-treatment. Outcome measures include: cancer threat appraisal; decision-related distress and bother from treatment side effects; involvement in decision making; satisfaction with health care; heath care utilisation; use of health care resources; and a return to previous activities.

**Discussion:**

The study will provide recommendations about the efficacy of early decision support to facilitate adjustment after prostate cancer. As well the study will identify men diagnosed with localised prostate cancer at risk of poorer long term psychosocial adjustment.

**Trial Registration:**

ACTRN012607000233426.

## Background

Internationally, prostate cancer is the second most common cancer diagnosed in men and the sixth most common cause of death [1]. With increasing rates of diagnosis and improved survival from prostate cancer the public health impact of prostate cancer is high. However, problematically, the benefits of early diagnosis and treatment of prostate cancer remain contentious. Prostate cancer is a heterogeneous disease and the risk of mortality from localised disease is difficult to quantify owing to the cancer's relatively slow growth rate. For example, 30–40% of all men aged over 50 years will be estimated to have histological evidence of prostate cancer, but of these only one in four men will develop clinically evident disease and only 1 in 14 will have disease that will prove lethal [2]. This means that many prostate cancers are not life threatening and treatment for men in this category may provide no potential benefit. On this basis, after the diagnosis of localised prostate cancer it is recommended that all men be advised of three possible treatment options at a minimum: close observation (no active treatment initially with intervention based on disease progression) or clinical monitoring; radiation therapy; or radical prostatectomy [3].

If watchful waiting reserves medical treatment for symptoms of prostate cancer, then although quality of life may be preserved, the opportunity for cure may be missed. For this reason, the more active surveillance strategy of close surveillance has evolved since a significant proportion of patients with low-risk prostate cancer do not progress in the short to intermediate term and that most of those who do progress are still able to have definitive therapeutic interventions without losing the likelihood of cure. By contrast, potentially curative treatments for localized prostate cancer such as surgery or radiation therapy have a range of deleterious side effects including impotence, urinary incontinence, bowel injury, urethral stricture and rarely death [4]. In addition, for the more aggressive forms of prostate cancer, there is a not-insignificant risk that, in spite of undergoing definitive treatment with curative intent with its associated morbidities, metastatic disease may be detected subsequently so that this unfortunate subset of patients experience locally-invasive treatments with seemingly little or no benefit. Therefore, men with localized prostate cancer are faced with a difficult treatment decision and decision-related distress is a highly salient aspect of their illness experience.

Most men prefer active involvement in their prostate cancer treatment decisions [5], however many find this difficult. First, as with most cancer treatments it is uncertain whether a cure will be attained. However, as outlined previously, for localised prostate cancer it is currently contentious whether any survival gain is likely. Second, side effects from treatment for localised prostate cancer are significant and so threat to the man's survival is balanced by threat to quality of life from changes to sexual, urinary and bowel functioning. Third, the risk probabilities for complications from treatment of localised prostate cancer are ambiguous with relevant local published outcome data often unavailable. For example, based mainly on North American data, risk estimates for impotence vary from 14% to 91% [4]. In uncertain and ambiguous situations, people find it difficult to choose [6]. Consequently, decision related distress is common for men after diagnosis with 63% of men reporting high decision-related distress that persists one year after treatment for 42% of all men [7]. As well, decision regret has been found to be associated with poorer quality of life three years after treatment for localised prostate cancer, with under-informed men at greater risk of regret [8].

Adding to this, the experience of the diagnosis and treatment of cancer is in itself a traumatic life event. The diagnosis of cancer poses a threat to the individual's survival and future hopes, to their physical and psychological sense of self, and to their social functioning. Elevated psychological distress such as anxiety and depression is common at initial diagnosis and during active treatments that are often accompanied by unpleasant side effects [9]. Patients' concerns may include fear and stigma associated with the diagnosis; fears about cancer recurrence; concerns about the physical effects of both the cancer and treatments; disturbances in self image, intimacy and sexuality; and disruption to daily activities [10].

Although many men report low psychological distress after diagnosis and treatment for prostate cancer, a substantial minority, between 14% and 38% of men, are highly distressed [7,11-14]. In addition, one third report moderate to high unmet supportive care needs in the domains of sexuality, psychological distress and treatment information up to five years after treatment [10]. Problematically, by contrast to women, men are less likely to seek help for psychological distress and are under-represented as clients to cancer support services. Thus, accessible support services for men with prostate cancer that are targeted to their specific concerns are critical. As well, psychosocial interventions that are targeted to those men at highest risk of distress are likely to be most efficacious [9].

A number of randomised controlled trials have been undertaken to assess the effectiveness of psychosocial interventions for men with localised prostate cancer. Interventions have included a range of therapeutic approaches such as psycho-education and peer discussion [15], uncertainty management [16], symptom management [17]; cognitive behaviour and stress management [18]. These studies have variously shown positive effects such as more stable employment, less sexual bother, improved general QOL and perceived stress management skills, improved uncertainty management, cognitive reframing and problem solving, and continence management and less cancer worry. However, none of these studies have targeted men at diagnosis when distress is highest, or addressed decision-related distress, or followed men past twelve months post-intervention. One randomised control trial has been reported for a self-efficacy information intervention delivered to men at diagnosis [19]. Men in the intervention group reported less anxiety and greater involvement in decision making, however the study involved a small convenience sample of 60 men with only a short six week follow up. Given the long lasting nature of treatment side effects such as erectile dysfunction and the potential for late decision-related distress and regret with implications beyond the patients themselves, longer term follow up of 2 and 3 years when treatment side effects have stabilised is essential.

In addition, there is considerable heterogeneity in the longitudinal course of adjustment of cancer patients as a whole and in regard to the different diagnostic groups to which they belong. Although the group means of functioning-related variables tend to shift in the more positive direction over time, many individual patients do not demonstrate such a pattern. Specifically, subgroups of patients may improve over time, by returning to baseline functioning, or may improve but not return to baseline functioning, or indeed may become worse over time [20]. After prostate cancer the trajectory of adjustment appears to differ for different subgroups of men: optimism and cancer threat appraisal are important in determining decision-related and psychological distress and global quality of life. In a prospective study in which 111 men were followed from diagnosis to one year post-treatment, optimism was a significant prospective and concurrent predictor of decision-related distress, with the effect mediated by proximal cancer threat appraisal [21]. Decision-related distress and optimism at diagnosis together predicted 45% of the variance of decision-related distress two months after treatment; decision-related distress at diagnosis and concurrent threat appraisal predicted 47% of this distress twelve months after treatment. Further two year follow up data found that, after adjusting for physical symptoms, optimism at diagnosis was a significant predictor of the level of psychosocial adjustment at each occasion of measurement but was not a predictor of the slope of psychosocial adjustment over time (unpublished data). Cancer threat appraisal was a partial and a full mediator of the effect of optimism on negative adjustment and positive adjustment, respectively. In turn, cancer threat appraisal predicted both the initial intercept and the slope of the trajectory of adjustment over time. Men whose cancer threat appraisal became more positive over time had a decreasing trajectory of negative adjustment. As men's cancer threat appraisal became more negative, their adjustment trajectory worsened. Previous approaches to decision support have been primarily utility based and have not considered the central role of cognitive appraisal in decision distress. We propose that including strategies that target negative cancer threat appraisals into a multi-component intervention that provides decision counselling as well as addressing the psychological and physical challenges associated with localised prostate cancer will be more effective.

## Methods/Design

### Study aims and hypotheses

The study aims first to assess the effectiveness of a decision support/psychosocial intervention in supporting men's decision making and improving their adjustment up to three years post-treatment when compared to usual care. Second, we will identify subgroups of men diagnosed with localised prostate cancer at risk of poorer long term psychosocial adjustment.

We hypothesise that up to three years after treatment for localised prostate cancer:

1. By contrast to men in usual care, men who receive the intervention will have a more positive cancer threat appraisal; less decision-related distress and bother from treatment side effects; more active involvement in decision making; greater satisfaction with health care; lower heath care utilisation; and a quicker and sustained return to previous activities.

2. Men with higher baseline decision-related and psychological distress will experience greater benefits from the intervention compared to men with lower baseline distress.

3. More optimistic men with more positive cancer threat appraisals will have an improved trajectory of adjustment by comparison to pessimistic men with a more negative appraisal.

### Intervention

Decision support is underpinned by the definition of an optimal decision as one that is both informed and in agreement with the person's values [22]. Existing evidence-based patient education materials and booklets are integrated into the intervention [23,24]. Working with these materials a nurse counsellor assists the patient to select out the information most relevant to his situation, his values and concerns, and also checks his comprehension of content in an ongoing educative process. Decision support is paired with the challenging of unhelpful cognitions; psycho-education about adjustment to prostate cancer; stress reduction techniques; and education and coaching about problem solving skills relevant to the side effects associated with prostate cancer treatments [25].

ProsCan for Men [26] is telephone delivered over five sessions by nurse counsellors guided by structured counseling protocols and supervised by a clinical psychologist with specialist training in oncology and clinical supervision. Supplementary self help worksheets that were developed specifically for this project are also utilised. Two sessions are delivered pre-treatment that focus on decision support, stress management and preparation for treatment. Two further sessions are delivered three and seven weeks after treatment focusing on adjustment to cancer, problem solving and coping with treatment side effects, with a fifth booster session five months after treatment focussing on treatment side effects and preparing for the future.

### Participants

Approximately 700 men newly diagnosed with prostate cancer (350 men in each condition) have been recruited into the ProsCan for Men study through committed participation by a large proportion of Queensland urologists. To recruit a broader and more diverse patient group the study was sited in the three cities of Brisbane, Townsville and Mackay that includes the geographic catchment areas of both Brisbane and environs and North Queensland. Men were referred to the project by their urologists if they were judged at the time of diagnosis to have localised prostate cancer suitable for treatment with curative intent and have no evidence of metastatic disease on scans and x-rays. Inclusion criteria were that the men must: (1) have been newly diagnosed with localised prostate cancer (2) be able to read and speak English (3) have no previous history of head injury, dementia or psychiatric illness and (4) have no other concurrent cancer. Informed written consent was obtained by study trained research nurses who contacted potential participants after referral to the project.

### Study integrity

Ethical approval has been obtained from the Queensland University of Technology Human Research Ethics Committee as well as the ethics committees of nine public hospitals in Queensland. The study design is guided by the CONSORT statement [27]. Randomisation to study condition was conducted following the completion of baseline assessment. Assessments are completed through telephone interviews and self-report pen and paper measures and project staff tracking assessments are blinded to condition. Randomisation occurred in blocks of 12, with each condition randomly generated 6 times within each block to ensure an unpredictable allocation sequence with equal numbers of couples in each group at the completion of each block. This sequence was undertaken by the project manager and concealed from investigators. Therapy is manualised with 25% of intervention calls recorded and reviewed to ensure treatment adherence. All analyses will be conducted on the basis of intention to treat.

### Materials

A series of previously validated and reliable self report measures are administered by mail to men in both the control and intervention groups at baseline, 2, 6, 12, 24 and 36 months after treatment. Brief single item screening measures for decision-related and psychological distress are used and will be cross validated to decisional conflict and psychological distress measures. Optimism is included as a predictor of adjustment and cancer threat appraisal as a moderator of intervention effect. Outcome variables are psychological distress; overall and domain specific QOL; decisional conflict; decision regret; involvement in decision making; satisfaction with and utilisation of health care. Disease variables (e.g. cancer grade, stage) are assessed though medical and cancer registry records review. Use of medical services and associated costs will be assessed through Medicare Australia records.

### Self report measures

#### Distress screening

The single item Distress Thermometer is widely used as a screening measure to assess global psychological distress [28]. This scale asks men how distressed they feel on a ten point scale. This scale has good sensitivity and specificity when used with a cut off point of > 5 [9]. As well, we have developed in our pilot work a novel single item five point scale to screen for high decision-related distress. This single item decision distress scale correlates highly with total Decisional Conflict Scale scores (r = .58, p < .005) [26] and decision status (r = .44, p < .05). A cut off of >2.5 indicates high decision distress associated with decision delay.

#### Optimism

Dispositional optimism is defined as generalised outcome expectancies where optimistic people have positive expectancies and pessimistic people have negative expectancies and is assessed by the Life Orientation Test-Revised (LOT-R [29]). Internal consistency for the LOT-R is very good. Dispositional optimism is predictive of psychological distress after a cancer diagnosis with more optimistic patients experiencing less distress [21].

#### Cancer threat appraisal

Men's cancer threat appraisal is measured with the Constructed Meaning Scale (CMS [30]) that assesses a person's cognitive construal over the week leading up to the time of assessment of the perceived consequences of their cancer diagnosis for their: a) sense of identity; b) interpersonal relationships; c) and generalised future prospects. The measure has good internal consistency. Positive appraisals of the meaning of the cancer diagnosis as assessed by the CMS are predictive of lower decision-related distress in men with prostate cancer [21].

#### Psychological distress

The Revised Impact of Events Scale (RIES [31,32]) is used to measure men's psychological distress. The RIES has 15 items and contains two subscales: intrusion and avoidance. Internal consistencies for the RIES subscales are good. Epping-Jordan [33] suggest that intrusion and avoidance are more sensitive measures of psychological distress after a cancer diagnosis than generalised distress measures. In men treated for localised prostate cancer intrusion and avoidance have been found to be related to poorer mental health outcomes [34].

#### Decision-related distress

For decision-related distress we assess both decisional conflict and decision regret. The Decisional Conflict Scale-Revised (DCS [21,35]) measures a person's perception of the difficulty involved in making a decision about medical treatments. The revised scale has 19 items covering decisional uncertainty, feeling uninformed, unclear about personal values, and unsupported in decision making, and perceptions of effective decision making. The DCS has been validated in a range of population groups and is sensitive to people making different health decisions, and to the effect of decision aids, with good internal consistency for the total scale. Health decisions assessed with this scale include treatment for localised prostate cancer. The Decision Regret Scale is a 5-item scale measuring distress or remorse after a health decision [36]. The scale has good internal consistency and has been validated in a range of populations including men considering prostate cancer treatment [36].

#### Desire for involvement in decision making

To assess desire for involvement in treatment decision making men were asked how they would prefer decisions about their prostate cancer treatment to be made on a five point Likert type scale from the doctor alone to you alone [37]. After treatment self report of actual involvement in decision making is assessed on this scale.

#### Subjective well being – satisfaction with life

Subjective well being is an important cognitive-judgemental psychological outcome from a stressful event that equates with global quality of life [38]. Subjective well being is measured with the generic five item Satisfaction with Life measure (SWL [39]). The internal consistency for SWL is good.

#### Treatment side effects

Urinary, sexual and bowel problems associated with prostate cancer treatments are measured by the symptom and bother subscales of the UCLA Prostate Cancer Index [40]. This 20 item measure includes subscales for each of the three problem domains of urinary, sexual and bowel functioning. Men respond with the frequency with which they experience symptoms and bother. Internal consistencies for these subscales are acceptable.

#### Health related Quality of Life

Health related quality of life is assessed with the SF-36 that is the most widely used QOL measure in the world with norms for the Australian general population available. The SF-36 [41] contains a mental health and physical health summary scale suitable to measure the impact of the intervention on patients' wellbeing. The SF-36 will also be used for translation into a health status measure for economic evaluation – the SF-6D. The SF-6D generates preference-based valuations of health states, allowing derivation of quality-adjusted life years.

#### Utilisation of health care

Data on health service utilisation will include hospitalizations, surgical procedures, diagnostic and imaging services, prescribed medications, GP and specialist visits, and other health professional visits. These will be measured using a combination of recommended valid and reliable methods [42]. Medicare Australia will provide service and cost data on GP services, drug use, diagnostic and imaging tests. Queensland Health will provide service and cost data on hospitalization and surgical procedures through the Queensland Hospital Admitted Patient Data Collection (and includes private and public hospital data). Data on emergency hospital visits and other outpatient visits will be abstracted from patient medical charts and valued using the Australian-Diagnostic Related Group cost estimates. The above health utilisation data will be collected by self-report questionnaires. Data collected by objective data methods will be verified by these self-reports where overlaps occur. Collectively, these data sources will give a comprehensive account on the typical health services used by our participants in the diagnosis and management of prostate cancer (and for all their health needs) during the study period and potential savings that may be attributable to the intervention.

#### Employment outcomes

Questions will be asked on pre-cancer and current employment status, time off work, returning to work, receipt of sickness and other benefits, unpaid work activities, changes in work role, consequences at work due to cancer, perceived work productivity, assistance from family or others, and carer's or partner's work patterns since the participant's cancer diagnosis.

### Statistical analyses

The study is a multivariate, two condition randomised controlled trial with repeated measures across time. The analysis of longitudinal differences in outcome will use two complementary statistical approaches: multilevel modelling (MLM) and growth mixture modelling (GMM) as applied to randomized preventative interventions by Muthén [43]. These procedures allow the testing of typical group level predictions such as Hypothesis 1 that men in the intervention group will have better psychosocial outcomes than the control group. However, they further permit the true assessment of Hypotheses 2 and 3 that deal with the impact of the intervention on heterogeneous subgroups of men. GMM is a latent variable technique that identifies subgroups of trajectories of change over time within a sample and allows the assessment of predictors of membership of trajectory groups, such as a high baseline distress group in the present proposal who are predicted to exhibit the strongest response of men in the intervention condition. GMM has the advantages of allowing use of all data points, which maximizes power to detect effects and reduces bias owing to missing data in longitudinal studies. Although power calculations are not well articulated for GMM, simulation studies suggest that the initial sample size of 350 per group will give at least 80% power to detect intervention effects of .5 (moderate) even with completion rates of 70% that are lower than the projected 85% [44]. As well, 350 per condition is sufficient to allow for the likely 2–3 subgroups of low to high risk men to be identified in the GMM.

Unlike previous approaches using ordinary least squares regression, MLM and GMM allow both the starting points and the rates of change in outcome variables for individual men to be parameters in the statistical models. The particular usage of MLM and GMM in the present application is for longitudinal effects where the number of assessments in time is a crucial parameter. Importantly, by adding two more assessment time points to the current RCT, the proposed study will allow analysis of both curvilinear change and of heterogeneity in response to intervention and the cancer experience. This will provide major insight into the likely long term impact of the intervention on men's health and well-being.

A cost-effectiveness analysis will determine if the intervention represents a good health investment. This involves the assessment of the efficacy data in conjunction with cost data collected on intervention resources, patient expenses, medical care utilization and producing incremental ratios of cost per quality-adjusted life year. The extended assessment time points will enhance the economic analysis because patient outcomes will be more comprehensive facilitating higher precision on the impact of prostate cancer on resource use.

## Discussion

This research will provide recommendations about the efficacy of tele-based nurse counselling to: facilitate more effective decision making about treatment; improve long term adjustment after prostate cancer; as well elucidating the potential economic value of the intervention. In addition, we will be able to validate approaches to identifying patients at high risk of poorer long term adjustment outcomes so that more in depth and intensive care can be directed to those patients with higher need [9]. It has been argued that future research into the effectiveness of psychosocial interventions in cancer should: be theory based and articulate the mechanisms of change; target patients at the point of highest distress and greatest need; include sexual adjustment as an outcome; use longitudinal designs with longer term follow up; and identify the moderators of treatment effect [45]. The current research addresses these recommendations and is the first of its kind internationally to do so for men with prostate cancer.

## Competing interests

The authors declare that they have no competing interests.

## Authors' contributions

SKC and JA developed the study concept and aims and initiated the project. MF, RAG, DN, SO and LG assisted in further development of the protocol. SC was responsible for drafting the manuscript. SC, JA and MF will implement the protocol and oversee collection of the data. All authors contributed to the final manuscript.

## Pre-publication history

The pre-publication history for this paper can be accessed here:


